# BioSentinel: Validating Sensitivity of Yeast Biosensors to Deep Space Relevant Radiation

**DOI:** 10.1089/ast.2022.0124

**Published:** 2023-05-22

**Authors:** Lauren C. Liddell, Diana M. Gentry, Rachel Gilbert, Diana Marina, Sofia Massaro Tieze, Michael R. Padgen, Kylie Akiyama, Kyra Keenan, Sharmila Bhattacharya, Sergio R. Santa Maria

**Affiliations:** ^1^ NASA Ames Research Center, Moffett Field, California, USA.; ^2^ Logyx LLC, Mountain View, California, USA.; ^3^ FILMSS/KBR, NASA Ames Research Center, Moffett Field, California, USA.; ^4^ Amyris, Inc., Emeryville, California, USA.; ^5^ Yale School of Medicine, New Haven, Connecticut, USA.; ^6^ Space Life Sciences Training Program, NASA Ames Research Center, Moffett Field, California, USA.

**Keywords:** *Saccharomyces cerevisiae*, Deep space, Space radiation, Biosensor, BioSentinel, CubeSat

## Abstract

With the imminent human exploration of deep space, it is more important than ever to understand the biological risks of deep space radiation exposure. The BioSentinel mission will be the first biological payload to study the effects of radiation beyond low Earth orbit in 50 years. This study is the last in a collection of articles about the BioSentinel biological CubeSat mission, where budding yeast cells will be used to investigate the response of a biological organism to long-term, low-dose deep space radiation. In this study, we define the methodology for detecting the biological response to space-like radiation using simulated deep space radiation and a metabolic indicator dye reduction assay. We show that there is a dose-dependent decrease in yeast cell growth and metabolism in response to space-like radiation, and this effect is significantly more pronounced in a strain of yeast that is deficient in DNA damage repair (*rad51*Δ) compared with a wild-type strain. Furthermore, we demonstrate the use of flight-like instrumentation after exposure to space-like ionizing radiation. Our findings will inform the development of novel and improved biosensors and technologies for future missions to deep space.

## 1. Introduction

In this new era of space exploration, astronauts will first return to the lunar surface and then eventually take the next giant leap to explore Mars. As we venture deeper into space for longer durations, we need to understand the biological risks that long-term spaceflight exposure can impose on organisms, and how these risks can be prevented or mitigated. Such deep space missions are expected to require significant biomedical and technological countermeasures to protect humans from the chronic radiation exposure they will encounter. Chronic exposure to galactic cosmic rays (GCRs), high-energy solar protons, and the possibility of large solar particle events poses the most significant health risks to humans, potentially increasing the risk of astronauts developing cancer as well as degeneration of the central nervous system, which can result in cognitive and behavioral impairment or neurological disorders (Chancellor *et al*., 2014; Takahashi *et al*., 2018). Currently, we cannot accurately simulate the deep space radiation environment in ground-based facilities. Thus, *in situ* experiments are critical to understand the effects of deep space on living organisms.

Current missions beyond low Earth orbit (LEO) are limited in the types of organisms they can use for biological research. The extended prelaunch periods, where biology remains loaded in the payload for multiple months under ambient or uncontrolled conditions, and long mission durations currently limit such voyages to model organisms that can remain dormant for prolonged periods. The budding yeast *Saccharomyces cerevisiae* is a prime candidate for deep space travel (Kachroo *et al*., 2015; Massaro Tieze *et al*., 2020; Santa Maria *et al*., 2020). Not only can cells remain viable despite spending years in a desiccated state, but yeasts also have a high molecular similarity to humans, sharing hundreds of genes essential for basic cell function. Despite the differences in genetic complexity and the simplicity of budding yeast (*i.e.*, *S. cerevisiae'*s genome is significantly smaller than the human genome thus a smaller target to genotoxic agents and its higher resistance to such agents), yeast and humans share many mechanisms involved in the response to DNA damage and in DNA repair, including the response to space-like ionizing radiation (IR).

Moreover, many genes involved in human diseases and carcinogenesis were first discovered and studied using budding yeast. The yeast intended for experiments during the BioSentinel mission will have been loaded on the launch vehicle for over 2 years by the time they have been rehydrated in space, further highlighting the value and suitability of this species to biological research in deep space. We previously optimized yeast survival for the BioSentinel mission by air drying cells in 10% trehalose, which acts as a defense against the stresses of desiccation (Coutinho *et al*., 1988). The methods for the long-term desiccation and storage of yeast cells in preparation for this mission were described previously (Santa Maria *et al*., 2020).

BioSentinel is 1 of 10 secondary payloads to launch on Artemis I—the first test launch of NASA's Space Launch System rocket and the Orion spacecraft. After a short lunar fly-by, and once it reaches its heliocentric orbit, BioSentinel will measure the response to the deep space environment using *S. cerevisiae* yeast cells and a microfluidic-based growth and metabolism detection system (Padgen *et al*., 2021). This will be compared with information provided by an onboard radiation sensor, and data obtained in LEO on the matching International Space Station (ISS) control unit, which was launched on the SpaceX-24 resupply mission on December 21, 2021. BioSentinel will measure the effects of the deep space environment, including high-energy radiation and microgravity, on biology over a minimum of 6 months.

In this study, we have conducted ground control experiments simulating the space-like radiation BioSentinel will encounter by utilizing IR sources, including the particle accelerator facilities at the NASA Space Radiation Laboratory (NSRL) at Brookhaven National Laboratory. In BioSentinel, after exposure to space radiation at varying doses, the desiccated cells will be rehydrated and their subsequent metabolism measured during the growth phase using the alamarBlue (aB) metabolic indicator dye (Santa Maria *et al*., 2020). Two diploid strains were selected for the mission, a wild-type (WT) strain and a mutant carrying a deletion of the *RAD51* gene (Santa Maria *et al*., 2020). While the WT serves as a control for cell health and normal DNA damage repair, the *rad51*Δ strain is defective in the repair of IR-induced damage and thus provides a more sensitive background for alterations to growth and metabolism. We show that *rad51*Δ cells display a dose-dependent reduction in metabolic activity and cell growth. These cells also display metabolic sensitivity to radiation doses as low as 10 cGy for both single ion and GCR simulations, which is similar to what astronauts are expected to encounter on a 1-year mission on the ISS, or an extended mission on a lunar operation or deep space habitat (Simonsen *et al*., 2020).

The establishment of these methods not only confirms their suitability for use in the upcoming BioSentinel deep space and LEO missions, but the procedures described in this study can help define methodologies for detecting a sensitive, biological response to space-like radiation that can be applied to future deep space missions.

## 2. Materials and Methods

### 2.1. Yeast strains

Standard genetic cross and tetrad dissection procedures were used to generate the WT (YBS71-1) and *rad51*Δ (YBS72-1) strains used in this study. Both strains are diploid prototrophic derivatives of the W303 background (*MATa/MATα ADE2 CAN1 HIS3 LEU2 TRP1 URA3*).

### 2.2. Yeast desiccation

The method to air-dry budding yeast cells for the BioSentinel mission has been described previously (Santa Maria *et al.*, 2020). Briefly, yeast from frozen glycerol stocks were grown on yeast extract-peptone-dextrose (YPD) agar (Y1500; Sigma-Aldrich) for 2–3 days at 30°C. Samples from freshly grown patches were then inoculated into 5 mL of liquid YPD (Y1375; Sigma-Aldrich) and grown on a rotating mixer at ambient temperature (∼23°C) for 7 days. Liquid cultures were pelleted and washed with sterile water, and the cell density was determined by hemacytometer counting. Cell suspensions were prepared to a final density of 1 × 10^7^ cells in 1 mL of 10% trehalose (T0167; Sigma-Aldrich). Ten microliter aliquots of the 1 × 10^7^ cell suspension (containing ∼10^5^ cells) were gently dispensed into the bottom edge of the wells of 96-well Stripwell™ microplates (9102; Costar^®^) or onto the sidewall of each microfluidic card well (Padgen *et al*., 2021).

Plates were sealed with Breathe-Easy^®^ gas permeable sealing membrane (Z380059; Sigma-Aldrich), and the cells were air dried in a 23°C incubator, 20–30% relative humidity, for 7 days. Yeast cells in fluidic cards were air dried inside sterile boxes at the same conditions before card sealing using a pneumatic press (Padgen *et al*., 2021).

### 2.3. IR exposures

In this study, IR exposures were performed by using a Cs^137^ gamma ray source at NASA Ames Research Center and accelerated particle beams at the NSRL at Brookhaven National Laboratory in New York. WT and *rad51*Δ cells dried in 10% trehalose in Stripwell microplates or inside microfluidic cards were exposed in both a desiccated state and liquid suspension. When exposed in liquid suspension, cells were rehydrated in Synthetic Complete (SC) growth medium containing 1 × aB metabolic indicator dye (DAL1100; Invitrogen) before each exposure (Santa Maria *et al*., 2020).

#### 2.3.1. Gamma radiation

WT and *rad51*Δ cells in Stripwell plates were first rehydrated with 100 μL 1 × aB+SC, incubated for ∼30 min, then gently pipette mixed before the exposures. Plates were then placed inside a Cs^137^ source chamber and exposed to 0, 0.5, 1, 2.5, 5, 10, 20, 30, 40, 50, or 60 Gy of gamma radiation. Once the exposures were completed, cell suspensions were transferred to clear 96-well nontreated polystyrene flat-bottom microplates (351172; Falcon^®^) for plate reading. All experiments were performed in triplicate.

#### 2.3.2. High-energy heavy ion and proton exposures

*rad51*Δ yeast cells in microwell plates in both a desiccated state and liquid suspension were placed in the beam line at NSRL and exposed to 0, 0.01, 0.1, 0.25, 0.5, 1, or 2.5 Gy of 300 MeV/n iron (Fe-56). WT cells were exposed to 0, 1, or 2.5 Gy of 300 MeV/n iron. After sample release, three wells of dried cells per condition were rehydrated with 100 μL 1 × aB+SC, incubated for 30 min, then gently pipette mixed. Cell suspensions were then transferred to clear 96-well microplates for plate reading.

Desiccated cells in microfluidic cards were exposed to 1 GeV protons (H-1) at 0 and 25 cGy at NSRL. Once the card radioactivity reached background levels, the samples were released from NSRL, ∼4 h after the proton exposures. The fluidic cards were then mounted on custom-made fluidic manifolds and filled with 1 × aB+SC medium using a peristaltic pump. Optical measurements were performed as described below.

#### 2.3.3. GCR simulation

Simplified 5-ion GCR simulations were conducted at NSRL. Simulations consisted of six sequential beams, including two proton and one helium energies, in addition to three heavy ion species (O-16, Si-28, and Fe-56) (Simonsen *et al.*, 2020). Yeast cells in suspension were exposed to 0, 0.01, 0.05, 0.1, 0.35, 0.5, or 1 Gy doses of GCR simulations. After sample release from NSRL, cell suspensions were transferred to 96-well microplates for plate reading. All experiments were performed in triplicate.

### 2.4. aB assay and data processing

Following irradiation (and after rehydration for samples exposed in a desiccated state), microplate reader optical measurements of yeast cells in 1 × aB+SC were taken once per hour on Molecular Devices SpectraMax 250/340 plate readers at 570 nm (detects concentration of pink aB), 600 nm (detects concentration of blue aB), and 750 nm (detects cell density or turbidity) for at least 48 h. Percent reduction of aB (or [*B*] below) was calculated for each experimental well using the following formula, modified from the manufacturer's manual to account for optical differences related to cell density (750 nm):

$$\left[B\right]=100-\left(\frac{A1-\left(O\times R1\right)-P\left(A2-O\times R2\right)}{\left(\left(1-\left(P\times B\right)\right)÷{\left[B\right]}_{0}\right)}\times 100\right)$$

where:

*A*1 = absorbance at 600 nm.

*A*2 = absorbance at 570 nm.

*O* = absorbance at 750 nm, representing optical density (OD) of the cells.

*R*1 = constant (ratio of cell density observed at 600 to 750 nm).

*R*2 = constant (ratio of cell density observed at 570 to 750 nm).

*P* = constant (ratio of pink-form aB observed at 600 to 570 nm).

*B* = constant (ratio of blue-form aB observed at 570 to 600 nm).

[*B*]_0_ = percent reduction of aB at time zero.

The data were plotted in Microsoft Excel. Note that percent reduction of aB in flight-like microfluidic cards was calculated using the same formula. However, the light emitting diode (LED) wavelengths measured in cards are 570, 630, and 850 nm. The values used for the constants *R*1, *R*2, *P*, and *B* were changed accordingly. We were unable to use the same wavelengths due to constraints of the SpectraMax 250/340 plate readers. Preliminary tests confirmed that aB reduction curves were not significantly different at these wavelengths (data not shown).

### 2.5. Flight-like microfluidic card experiments

Once sealed, the cards containing the desiccated yeast cells were exposed to 1 GeV protons at NSRL. After filling with 100 μL 1 × aB+SC medium, optical measurements were performed by using flight-like LED emitter and detector boards (Padgen *et al*., 2021). Each fluidic card contains 16 microwells, and each microwell is illuminated by 3 LED lights, with optical measurements performed every 2 min. Similar to the wavelengths utilized by the microplate readers, a 570 nm LED measures the appearance of the reduced pink form of aB, a 630 nm LED measures the disappearance of the oxidized blue form, and an 850 nm LED measures optical density or turbidity (Padgen *et al*., 2021).

### 2.6. Statistical analyses

To assess the metabolic effects of radiation, triplicate values of percent reduction of aB at each radiation dose were averaged and plotted over time with standard deviation error bars. To compare differences in aB reduction across samples, we used the threshold slope, which is the slope from 10% reduction in aB to 85% reduction, as a first parameter of interest (Supplementary Methods). A value of 10% was chosen because detectable cell growth does not typically occur until aB reaches ∼10% or 5–7 h. The 85% value is based on the point at which all samples within the experiment have reached a common maximum threshold of aB reduction. For the fluidic card analysis, a top threshold of 70% was used since there were many replicates that did not reach higher than 70% reduction in aB. Slope values were compared between each radiation dose condition using the Student *t*-test (Supplementary Methods). A *p*-value of <0.05 denotes radiation sensitivity in comparison with the 0 Gy (unexposed) dose or a significant difference in metabolic response to radiation between doses. Statistical analyses were completed in Microsoft Excel, and graphs were created using GraphPad Prism.

## 3. Results

### 3.1. Testing sensitivity of yeast cells to IR using a redox indicator dye

**FIG. 1.**
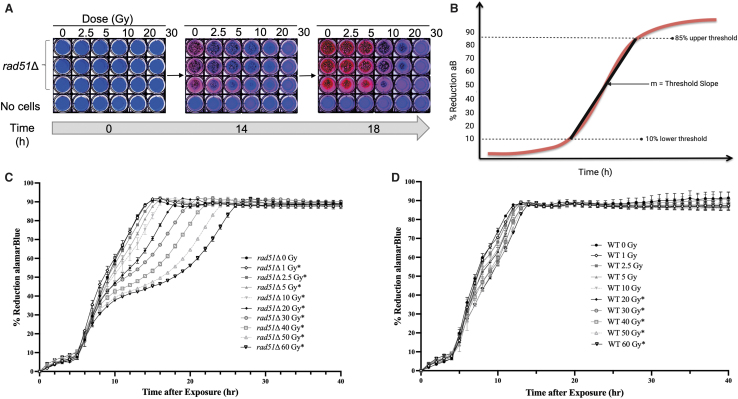
Testing sensitivity of yeast cells to gamma radiation using the aB reduction assay. **(A)** Color change transition of the aB dye over time after exposure of *rad51*Δ yeast cells in 1 × aB+SC medium suspension to increasing doses of Cs^137^ gamma radiation at 0, 2.5, 5, 10, 20, or 30 Gy. **(B)** Diagram showing how the threshold slope for the aB reduction assay is calculated. aB reduction profiles of **(C)** *rad51*Δ and **(D)** WT yeast cells exposed in 1 × aB+SC medium to increasing doses of gamma IR. Sensitivity to gamma IR was assessed by comparing threshold slopes of each dose to the unirradiated controls and through the Student *t*-test (* denotes *p* < 0.05). All experiments were performed in triplicate. aB, alamarBlue; IR, ionizing radiation; SC, synthetic complete; WT, wild type.

Because the BioSentinel free flyer CubeSat is not returning to Earth, it is critical that the aB kinetic and growth measurements be sensitive enough to remotely detect differences to low doses of space radiation, even after spending a prolonged period of time in a desiccated state. After WT and *rad51*Δ yeast were desiccated and rehydrated with SC medium containing aB, they were exposed in liquid suspension to increasing doses of gamma radiation (Fig. 1). An example of how the aB dye changes color over time after exposure of *rad51*Δ cells to gamma IR is shown in Fig. 1A. When comparing the percent reduction of aB over time (threshold slope, Fig. 1B), we found that the *rad51*Δ mutant strain was sensitive to gamma radiation doses as low as 1 Gy (*p* = 0.039), and that there is an increasing dose-dependent sensitivity at 2.5 Gy (*p* = 0.033), 5 Gy (*p* = 0.014), 10 Gy (*p* = 0.003), 20 Gy (*p* = 0.0006), 30 Gy (*p* = 0.0002), 40 Gy (*p* = 0.0001), 50 Gy (*p* = 0.00008), and 60 Gy (*p* = 0.00007) (Fig. 1C).

For the WT strain, sensitivity to gamma radiation was lower than the *rad51*Δ mutant, as this strain showed a dose-dependent sensitivity starting at 20 Gy (0.021) and at subsequent doses of 30 Gy (0.001), 40 Gy (*p* = 0.002), 50 Gy (*p* = 0.002), and 60 Gy (*p* = 0.001) (Fig. 1D). Using a regression analysis, we confirmed that the aB redox indicator dye is strongly correlated with cell growth (OD measurement at 750 nm) in both strains at both high radiation of 60 Gy (*r* = 0.9624 for WT and *r* = 0.9252 for *rad51*Δ) and no radiation (*r* = 0.968 for WT and *r* = 0.9453 for *rad51*Δ) (Supplementary Fig. S1).

### 3.2. Testing sensitivity of yeast cells to IR in suspension versus in desiccated form

**FIG. 2.**
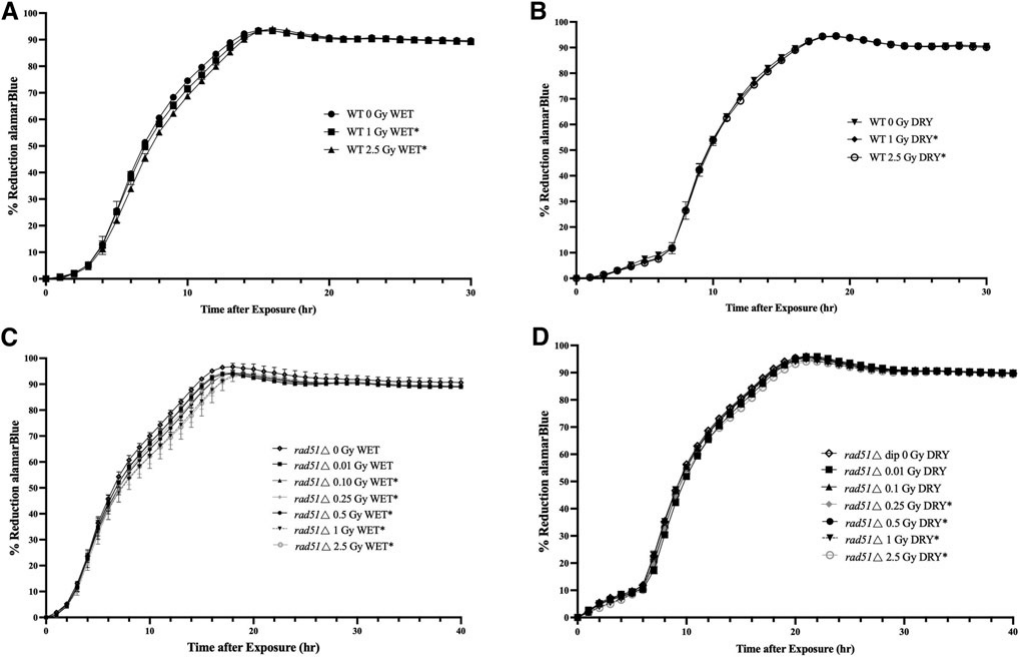
Sensitivity of yeast cells exposed to high-energy iron particles in suspension and in desiccated form. aB reduction profiles of WT and *rad51*Δ yeast cells exposed to 300 MeV/n iron in either liquid suspension (1 × aB+SC growth medium) **(A, C)** or in desiccated state **(B, D)** at the NSRL. Sensitivity was assessed by comparing threshold slopes of each dose to the unirradiated controls and through the Student *t*-test (* denotes *p* < 0.05). All experiments were performed in triplicate. NSRL, NASA Space Radiation Laboratory.

In BioSentinel, the yeast cells will be exposed to space radiation primarily in a desiccated state. Therefore, it is important to demonstrate that the yeast cells are sensitive to space-relevant IR not only in liquid suspension but also in desiccated form. In a previous study, both WT and *rad51*Δ strains exhibited a dose-dependent response to high-energy protons in desiccated form (Santa Maria *et al*., 2020). In this study, we exposed them to increasing, low doses of 300 MeV/n iron particles at NSRL in both desiccated state and in liquid suspension (Fig. 2). Both groups were monitored by the metabolic aB assay over a 24-h period. As expected, the *rad51*Δ strain displayed sensitivity in both states (Fig. 2C, D), showing a change in aB reduction in the liquid suspension starting at 0.1 Gy (*p* = 0.021), then further sensitivity at 0.25 Gy (*p* = 0.001), 0.5 Gy (*p* = 0.0002), 1 Gy (*p* = 0.02), and 2.5 Gy (*p* = 0.0001) (Fig. 2C) and in the desiccated exposure state at 0.25 Gy (*p* = 0.01), 0.5 Gy (*p* = 0.03), 1 Gy (*p* = 0.033), and 2.5 Gy (*p* = 0.0001) (Fig. 2D).

WT cells were sensitive to both 1 Gy (*p* = 0.036) and 2.5 Gy (*p* = 0.043) in liquid suspension (Fig. 2A), and they were similarly sensitive to both 1 Gy (*p* = 0.025) and 2.5 Gy (*p* = 0.005) when cells were exposed, while in the desiccated state and rehydrated for aB measurements (Fig. 2B).

In summary, our aB metabolic assay is sensitive enough to detect changes in the aB metabolic profile in response to space-relevant IR and doses relevant for future deep space missions. Importantly, the assay is also sensitive enough to detect metabolic changes of yeast exposed to radiation while in both liquid suspension as well as in the desiccated state, which is relevant for the BioSentinel mission.

### 3.3. Testing flight strains and the aB assay in response to GCR simulations

**FIG. 3.**
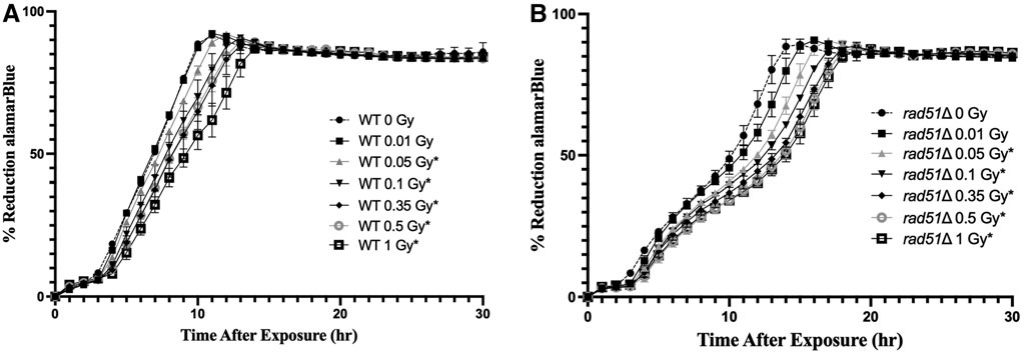
Sensitivity of BioSentinel's flight strains to GCR simulations. WT **(A)** and *rad51*Δ **(B)** yeast cells were exposed to increasing doses of 5-ion GCR simulations at the NSRL. Exposures were performed with cells in 1 × aB+SC medium suspension in microwell plates. Sensitivity was assessed by comparing threshold slopes of each dose to the unirradiated controls and through the Student *t*-test (* denotes *p* < 0.05). All experiments were performed in triplicate. GCR, galactic cosmic ray.

During the development of the BioSentinel mission, GCR simulations became available to the scientific community at NSRL. Although we cannot exactly reproduce the radiation environment BioSentinel yeast will encounter during its mission beyond LEO, the GCR simulations provide the closest proxy available on the ground (Simonsen *et al.*, 2020). WT and *rad51*Δ strains were exposed to increasing doses of GCR simulations in 1 × aB+SC liquid suspension and measured growth and aB reduction for 40 h (Fig. 3). The WT strain showed sensitivity to GCR simulations at 0.05 Gy (*p* = 0.0129), 0.1 Gy (*p* = 0.009), 0.35 Gy (*p* = 0.0001), and 1 Gy (*p* = 0.0002) (Fig. 3A). Similarly, *rad51*Δ cells were sensitive at 0.05 Gy (*p* = 0.009), 0.1 Gy (*p* = 0.004), 0.35 Gy (*p* = 0.002), 0.5 Gy (*p* = 0.0018), and 1 Gy (*p* = 0.001) (Fig. 3B). These promising results indicate that both the WT and *rad51*Δ strains are sensitive to low doses of space-like radiation.

### 3.4. Testing sensitivity of BioSentinel flight strains using flight-like hardware

**FIG. 4.**
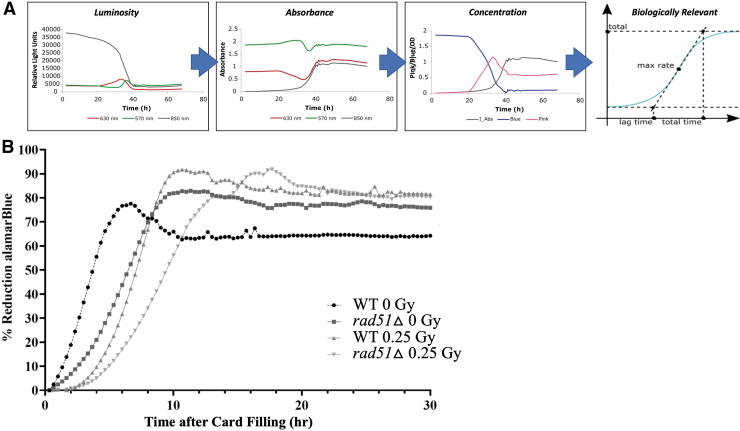
Sensitivity of BioSentinel strains to high-energy protons in flight-like microfluidic cards. **(A)** The data processing pipeline shows how data are processed from raw optical initial luminosity to biologically relevant measurements. Raw optical data are first downloaded from optical reading devices as luminosity (relative light units). Then, data points are converted to absorbance and concentration of the oxidized blue form and reduced pink form of the aB dye in addition to optical density or cell turbidity. The data sets are further processed to generate biologically relevant information, such as lag time, growth rate, cell duplication time, etc. **(B)** *rad51*Δ cells exposed in desiccated state inside a microfluidic card show sensitivity to 0.25 Gy of space-relevant 1 GeV protons compared with unirradiated control (*p* = 0.0008). On the other hand, WT cells show no significant sensitivity under the same conditions. After exposure and subsequent radioactive decay, the cards were filled with 1 × aB+SC medium and optical measurements performed using flight-like optical instrumentation. Sensitivity was assessed by comparing threshold slopes and through the Student *t*-test. Data points represent the average of eight replicates per strain.

To complete our validation experiments, the next step was to test both WT and *rad51*Δ strains in a more flight-like environment—exposed in desiccated state inside microfluidic cards using a flight optical detection system (Fig. 4). Unlike in 96-well plates, where the aB metabolic assay can be run in a standard microplate reader, cell activity in fluidic cards is monitored by using measurements taken with LED emitter and photodiode detector boards housed inside a 3D-printed case, which is part of the custom-made ground support equipment (GSE) tools we use to perform flight-like experiments in the laboratory. Importantly, these GSE optical readers contain the same electronic components found inside the BioSentinel flight units, including the three LED lights we will use in deep space (Ricco *et al*., 2020; Padgen *et al*., 2021). Thus, these ground control tests ensure that the aB assay and the payload instrumentation are sensitive enough to detect radiation-dependent metabolic changes in deep space.

There are some minor differences in how the GSE hardware records optical data compared with the plate readers used in previous experiments. Rather than reporting absorbance, which is the log-transformed ratio of light transmitted through a sample, the flight instrument reports the light transmitted (brightness) of each of the three measured wavelengths directly. These measurements should follow a certain pattern over time as the yeast grow, metabolize, and reduce the aB after radiation exposure (Fig. 4A). The red 630 nm LED signal should increase at first since the blue (oxidized) form of aB is disappearing (equivalent to a decrease in absorbance), and then it should decrease as the pink form of aB starts to appear (equivalent to an increase in absorbance). The green 570 nm LED signal should decrease as the pink (reduced) aB form starts to appear; then, as the aB continues to be reduced and becomes colorless, the green signal should increase again, generating a “double-hump” effect.

Finally, the near-infrared LED readings at 850 nm should start to decrease as the cells grow, indicating that cell density and turbidity is increasing. As cell density gets higher toward the end of the growth cycle, all three wavelengths will start to decrease together, as the turbidity becomes so high that it starts to block all three wavelengths of light.

For this experiment, we exposed desiccated yeast inside fluidic cards to 1 GeV protons at NSRL (Fig. 4B). Similar to what we see in the above results using microwell plates and using the same threshold slope analysis after converting brightness measurements to absorbance, the experiments in microfluidic cards show that the *rad51*Δ strain is sensitive to 25 cGy protons compared with both the unirradiated WT (*p* = 0.0029) and *rad51Δ* (*p* = 0.0008) strains. The exposed WT strain, on the other hand, is not significantly different compared with its unirradiated control (*p* = 0.119). These results indicate that, in flight-like conditions where the yeast cells are exposed to radiation while in a desiccated state and rehydrated later for measurement of metabolic activity, we see significant changes in sensitivity to radiation in *rad51*Δ cells at a level that the BioSentinel free flyer hardware will be able to detect.

## 4. Discussion

In the current study, we show that the aB assay is sensitive enough to measure metabolic changes in response to even small doses of space-relevant IR using the *S. cerevisiae* strains that will be employed in the BioSentinel deep space mission. We validated the assay itself by performing initial experiments using relatively high doses of gamma radiation and yeast in liquid suspension, and we further established that the strains preserve sensitivity to high-energy IR in both a desiccated form and liquid suspension. We then showed that the aB assay detects changes in response to space-like GCR simulations. Lastly, we demonstrated that the yeast is sensitive to radiation exposure while loaded into flight-like hardware and exposed while desiccated, which is similar to spaceflight conditions.

Importantly, the yeast samples in BioSentinel will be exposed to space radiation mostly in a desiccated state. Although we have observed a reduced response in desiccated cells compared with cells in liquid suspension, we have consistently observed a significant dose-dependent response in desiccated cells when using a variety of IR sources, including gamma rays, protons, high-energy heavy particles, and GCR simulations (Santa Maria *et al*., unpublished data). This is to be expected, considering the reduced number of water molecules in desiccated samples and, thus, a reduction in the production and exposure to free radical species that can damage cell membranes, lipids, DNA, etc. (Santa Maria *et al*., 2020). Taken together, these data demonstrate confidence in our approach for collecting and analyzing data from the upcoming BioSentinel deep space mission, which will be compared with data obtained on the ground and in LEO on the ISS.

The methods described in this study are meant as a proof of concept for the analysis of metabolic activity and growth of yeast in the presence of the aB redox dye. While we have chosen to show only one of the data processing tools (*i.e.*, threshold slope) as our initial proof-of-concept statistical test, other parameters of the curve analysis can be compared, such as the lag time before exponential growth or metabolic activity start, maximum rate of reduction, total time to peak reduction, yeast duplication rates, and the less frequently studied change of aB from pink to colorless form. We are currently investigating and optimizing parameters for the analysis of flight cell growth and aB reduction in yeast; however, it is important to note that the optimal parameters may differ depending on the application, experiment, or biological organism.

The aB method described in this study can only detect general changes in metabolism and growth in a cell population. The method is inadequate, however, with regard to specific metabolic pathways triggered by space radiation or the type of cell and DNA damage that triggers such response. To best inform future missions beyond LEO, it is essential to understand which molecular mechanisms are affected by the deep space environment, including both reduced gravity and space radiation. Exposure of cells to IR leads to the immediate formation of free radicals, which, despite their transient nature, can react with DNA, lipids, and cell membranes, often causing damage. In addition, intercellular metabolic redox reactions can be affected by IR-induced free radical formation and can remain perturbed for minutes, hours, or even days (Kohen and Abraham, 2002; Spitz *et al.*, 2004). This is particularly problematic since redox reactions run important cell processes, such as cellular respiration. Monitoring IR-dependent changes in the basic aB time-course reduction profile is only a starting point in understanding which cellular processes are affected by IR.

By understanding the redox potential of each form of aB in cellular respiration and then complementing this information with metabolic and molecular tests and modeling, we can begin to identify potential molecular mechanisms that are activated by space radiation. Measuring which of the aB-dependent metabolic processes are delayed after radiation exposure in our yeast cells can provide us with initial insight as to which molecular mechanisms are affected. These experiments can be validated in future ground studies, especially since we have now established methods by which we can perform preliminary analyses using GCR simulations or other simulation approaches for space-like conditions.

Future missions to LEO and beyond will have the capacity to carry a higher number of yeast strains with genetic defects in a variety of cellular pathways, including cells defective in DNA damage repair and stress response. This strategy, in addition to extensive ground research, will allow us to pinpoint specific mechanisms involved in the response to the deep space environment. One such effort is NASA's Lunar Explorer Instrument for space biology Applications (LEIA) mission, which will launch in 2026 and land on the lunar surface by way of a Commercial Lunar Payload Services lander. The LEIA mission will study the effect of the lunar radiation environment and partial gravity in *S. cerevisiae*, and it will use a variation of the microfluidic system originally developed for BioSentinel. As observed in our experiments, strains defective in the repair of damage caused by IR can have other phenotypes, including slower growth and metabolic rates when compared with WT (Santa Maria *et al.*, 2020). In preparation for LEIA, we are planning a series of ground validation studies that will include the BioSentinel strains with the intent to validate that the sensitivity observed is due to an actual cell response to IR and not to strain-specific phenotypes.

These initial investigations have provided the following preliminary conclusions in preparation for our flight mission beyond LEO. First, the shift in the aB metabolic response is dependent on the degree of exposure to IR. The higher the radiation dose, the further the reduction profile is shifted or delayed. Second, the longer the yeast cells are desiccated, the further the metabolic curve is shifted to the right. In other words, the older the cells are, the longer the delay in the aB metabolic reduction profile.

As we see it, there are a few potential mechanisms driving this shift in aB reduction, including (1) potentially fewer surviving cells that could result in a lag in the growth and reduction of aB over time, (2) an additional damage-induced lag period as cells react to radiation exposure (*e.g.*, by signaling DNA damage response mechanisms or temporarily halting cell division) before the cells return to normal activity, or (3) exposure to radiation causing permanent changes to the cell line and changing the growth kinetics (*e.g.*, generation time) entirely. As it stands, the aB assay alone is not sufficient to distinguish between these possibilities, and further testing is required to determine whether one or more are responsible. These preliminary observations have helped us gauge what to expect for an aB reduction timeline for flight and to fine-tune our science mission operations (*i.e.*, the number of fluidic cards activated per time point).

Designing biological experiments for deep space presents many unique challenges. Not only does an organism need to survive the constraints of a long-duration mission, but it must also be adaptable to the uniquely designed hardware, in this case the specialized microfluidic cards and manifolds. The combined effect of irradiating desiccated samples inside microfluidic cards demonstrates just one of the challenges of designing and conducting a space experiment. Each of the microfluidic cards built for flight is cost and labor intensive to produce, which limits the total number of cards available for ground testing and validation. Accordingly, most of our preliminary long-term desiccation, viability, and radiation tests were performed in standard microwell plates.

Studies along the lines of the present study highlight the use of model organisms like *S. cerevisiae* to investigate the biological response to the space environment in preparation for future crewed missions beyond LEO. Importantly, we have demonstrated the sensitivity of budding yeast and the aB reduction assay to low space-relevant doses of IR (Simonsen *et al*., 2020), and have developed instrumentation capable of supporting living organisms in free deep space missions (Massaro Tieze *et al.*, 2020; Santa Maria *et al.*, 2020; Padgen *et al*., 2021).

## 5. Conclusion

Our preliminary ground experiments with dormant *S. cerevisiae* cells in a desiccated state and exposure to space-relevant IR doses and types (gamma, protons, heavy ion, GCR simulations) have allowed us to define the aB assay for a biology mission beyond LEO. Future ground studies in support of new missions to deep space and the lunar surface will help to build upon the aB metabolic assay and attain deeper insight into those molecular mechanisms that are activated by space radiation. Finally, we hope that the scientific data we receive from the BioSentinel deep space CubeSat will help to inform and motivate the next generation of human explorers on the Moon and beyond and help train new generations of scientists and engineers.

## Supplementary Material

Supplemental data

Supplemental data

## Notes

Associate Editor: Petra Rettberg

## Abbreviations Used

aB: alamarBlue
GCR: galactic cosmic ray
GSE: ground support equipment
IR: ionizing radiation
ISS: International Space Station
LED: light emitting diode
LEIA: Lunar Explorer Instrument for space biology Applications
LEO: low Earth orbit
NSRL: NASA Space Radiation Laboratory
OD: optical density
SC: synthetic complete
WT: wild type
YPD: yeast extract-peptone-dextrose
