# Supplementary material for: BioSentinel: Validating Sensitivity of Yeast Biosensors to Deep Space Relevant Radiation
Source: Astrobiology. 2023 May 22;23(6):648–56. doi: 10.1089/ast.2022.0124 (PMC10254971; doi:10.1089/ast.2022.0124)
Supplement: Supplemental data [file Suppl_FigS1.pdf]

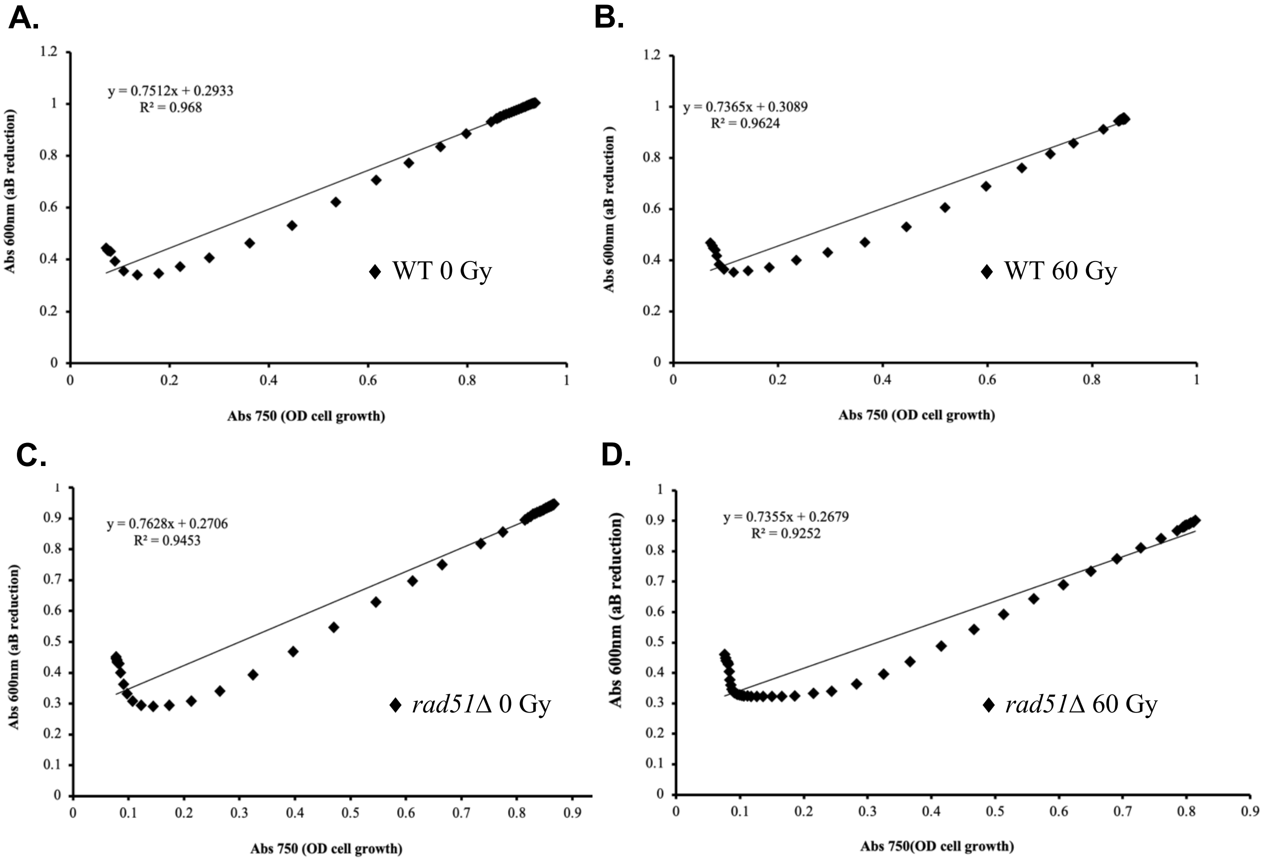

SUPPL. FIG. 1. alamarBlue (aB) reduction and cell growth regression analyses. Regression analysis shows strong correlation between aB redox dye reduction (600-nm absorbance) and cell growth (750-nm absorbance) in both wild type (WT) **(A-B)** and *rad51*Δ **(C-D)** cells exposed to 0 (r=0.968 for WT and r=0.9453 for *rad51*Δ) and 60 Gy (r=0.9624 for WT and r=0.9252 for *rad51*Δ) of Cs^{137} gamma radiation. All experiments were performed in triplicate.
