# Supplementary material for: BioSentinel: Validating Sensitivity of Yeast Biosensors to Deep Space Relevant Radiation
Source: Astrobiology. 2023 May 22;23(6):648–56. doi: 10.1089/ast.2022.0124 (PMC10254971; doi:10.1089/ast.2022.0124)
Supplement: Supplemental data [file Suppl_Data.pdf]

Supplementary Methods

Threshold slope analyses

To compare differences in alamarBlue (aB) reduction across samples, the threshold slope was used, which is the slope from 10% reduction in aB to 85% reduction, as a first parameter of interest. 10% was chosen because detectable cell growth does not typically occur until aB reaches approximately 10% or 5-7 hours. The 85% value is based on the point at which all samples within the experiment have reached a common maximum threshold of aB reduction. For the fluidic card analysis, a top threshold of 70% was used since there were many replicates that did not reach higher than 70% reduction in aB.

Slope values were calculated as follows:

For microwell plate analyses:

$$\text{Slope (m)} = \frac{(85\% - 10\%)}{(\text{time at } 85\% \text{ aB reduction } - \text{ time at } 10\% \text{ aB reduction})}$$

For fluidic card analyses:

$$\text{Slope (m)} = \frac{(70\% - 10\%)}{(\text{time at } 70\% \text{ aB reduction } - \text{ time at } 10\% \text{ aB reduction})}$$

Slope values were compared between each radiation dose condition using the Student's *t*-test. A *p* value of less than 0.05 denotes radiation sensitivity in comparison with the 0 Gy (unexposed) dose or a significant difference in metabolic response to radiation between doses. Statistical analyses were completed in Microsoft Excel, and graphs were created using Graphpad Prism.

FIG. 1 Threshold slopes and p values

FIG. 1C Threshold slopes (gamma IR WT)			
**Dose**	**Average Slope**	***t*-test**	***p* value**
0 Gy	9.963	N/A	N/A
0.01 Gy	10.661	-2.1450112	0.04926587
0.05 Gy	10.583	-1.5815447	0.09445663
0.1 Gy	10.607	-1.7817932	0.07468591
0.35 Gy	10.561	-1.1677623	0.15388073
0.5 Gy	10.027	-0.1584774	0.4408799
1 Gy	10.057	-0.247104	0.4084962
2.5 Gy	9.684	0.51498036	0.31685878
5 Gy	9.239	1.94801739	0.06161867
10 Gy	9.452	0.98280264	0.19067393
20 Gy	8.815	2.96415155	0.02069179*
30 Gy	7.773	6.11698183	0.00180843*
40 Gy	8.045	5.86159186	0.00211428*
50 Gy	8.008	5.74344376	0.00227714*
60 Gy	7.964	6.22411069	0.00169645*

FIG. 1D Threshold slopes (gamma IR *rad51*∆)			
**Dose**	**Average Slope**	***t*-test**	***p* value**
0 Gy	9.188	N/A	N/A
0.01 Gy	8.439	1.48132898	0.10632016
0.05 Gy	8.593	1.33677301	0.12613072
0.1 Gy	8.856	0.69851342	0.26166792
0.35 Gy	8.602	1.28485071	0.13410018
0.5 Gy	8.677	1.13223169	0.16040471
1 Gy	8.125	2.348665	0.03931497*
2.5 Gy	8.082	2.48605847	0.0338849*
5 Gy	7.67	3.40625441	0.01356034*
10 Gy	6.827	5.14717384	0.00337882*
20 Gy	5.523	8.17608917	0.00060929*
30 Gy	4.376	10.4731782	0.00023489*
40 Gy	3.861	11.9469305	0.00014063*
50 Gy	3.222	13.443614	8.8553E-05*
60 Gy	2.817	14.2712649	7.0014E-05*

FIG. 2 Threshold slopes and p values

**FIG. 2A Threshold slopes (iron WT WET)**			
**Dose**	**Average Slope**	***t*-test**	***p* value**
0 Gy	9.161	N/A	N/A
1 Gy	8.373	2.41756279	0.03647756*
2.5 Gy	8.308	2.26204761	0.0432422*

**FIG. 2B Threshold slopes (iron WT DRY)**			
**Dose**	**Average Slope**	***t*-test**	***p* value**
0 Gy	10.092	N/A	N/A
1 Gy	9.243	2.76057897	0.02540971*
2.5 Gy	8.905	4.60243659	0.00500625*

**FIG. 2C Threshold slopes (iron *rad51*Δ WET)**			
**Dose**	**Average Slope**	***t*-test**	***p* value**
0 Gy	6.767	N/A	N/A
0.01 Gy	6.402	1.37094377	0.12114027
0.1 Gy	6.413	2.9052617	0.02194295*
0.25 Gy	6.157	5.98063555	0.0019644*
0.5 Gy	5.894	9.95593909	0.00028585*
1 Gy	5.619	2.9838404	0.02029221*
2.5 Gy	5.424	11.7821102	0.00014848*

**FIG. 2D Threshold slopes (iron *rad51*Δ DRY)**			
**Dose**	**Average Slope**	***t*-test**	***p* value**
0 Gy	7.137	N/A	N/A
0.01 Gy	7.074	0.32940935	0.37918673
0.1 Gy	7.0006	1.4087798	0.11584163
0.25 Gy	7.413	-3.0311812	0.01936782*
0.5 Gy	7.293	-2.4680732	0.03454477*
1 Gy	6.817	2.48777659	0.03382262*
2.5 Gy	6.444	11.1593617	0.00018351*

FIG. 3 Threshold slopes and p values

**FIG. 3A Threshold slopes (GCRsim WT)**			
**Dose**	**Average Slope**	***t*-test**	***p* value**
0 Gy	11.475	N/A	N/A
0.01 Gy	11.755	-1.5071339	0.10312713
0.05 Gy	10.78	4.81837577	0.00426655*
0.1 Gy	9.824	3.35849647	0.01417218*
0.35 Gy	9.284	4.99121446	0.00376858*
0.5 Gy	9.414	9.09024996	0.00040604*
1 Gy	8.031	10.1515876	0.00026509*

**FIG. 3B Threshold slopes (GCRsim *rad51*Δ)**			
**Dose**	**Average Slope**	***t*-test**	***p* value**
0 Gy	6.582	N/A	N/A
0.01 Gy	5.999	1.81942401	0.07148637
0.05 Gy	5.489	3.70191665	0.01040026*
0.1 Gy	5.216	5.05142361	0.00361193*
0.35 Gy	4.996	5.96640498	0.00198161*
0.5 Gy	4.939	5.85933146	0.00211726*
1 Gy	4.79	6.42112146	0.00151191*

FIG.4 Threshold slopes and p values

**FIG. 4B Threshold slopes (protons WT)**			
**Dose**	**Average Slope**	***t*-test**	***p* value**
0 Gy	15.249	N/A	N/A
0.25 Gy	13.629	-1.233	0.1188172

**FIG. 4B Threshold slopes (protons *rad51*Δ)**			
**Dose**	**Average Slope**	***t*-test**	***p* value**
0 Gy	12.696	N/A	N/A
0.25 Gy	8.56	3.969	0.0008014*
